# Decontamination practices of paediatric crowns: a systematic review

**DOI:** 10.1007/s40368-022-00714-w

**Published:** 2022-05-24

**Authors:** T. Hogerheyde, L. J. Walsh, S. Zafar

**Affiliations:** School of Dentistry, The University of Queensland, 288 Herston Road, Herston, Brisbane, QLD 4006 Australia

**Keywords:** Paediatric, Preformed crown, Decontamination, Sterilisation, Disinfection

## Abstract

**Purpose:**

A systematic review was conducted into paediatric crown decontamination practices using the Preferred Reporting Items for Systematic Reviews and Meta-analyses guidelines.

**Method:**

After database retrieval using predefined search terms, two reviewers independently applied the selection criteria, extracted the data, and assessed for risk of bias. From 92 potentially eligible studies, 10 were included for analysis.

**Results:**

Steam sterilisation (autoclaving) was used as the gold standard for reducing biological contaminants on paediatric crowns across in vivo studies. However, autoclaving was associated with microstructural changes such as crazing and contour alterations. Furthermore, several tooth coloured crowns were liable to undergo colour changes from steam sterilisation.

**Conclusion:**

Ambiguous manufacturer guidelines on protocols for reprocessing and reuse after biological exposure raises concerns regarding cross contamination and leaves practitioners open to potential litigation. A better understanding of the compatibility of paediatric crowns and decontamination practices is needed. More research into alternative chairside technologies that use low temperature, such as hydrogen peroxide gas plasma sterilisation, is warranted.

## Introduction

Early childhood caries is a health epidemic that disproportionately affects the developing world and low socioeconomic communities (Çolak et al. 2013). Oral disease (primarily dental caries) in Australia accounts for 8.1% of non-fatal diseases for children aged 5–9, with numbers higher in indigenous children (Australian Institute of Health and Welfare 2021). A population-based study of dental caries noted children aged 5–10 had 1.5 decayed, missing, and filled deciduous teeth. Moreover, the rate of potentially preventable hospitalisations of children aged 0–14 years in 2017–2018 due to dental conditions was 17 per 1000 population. Due to high caries rate in children, the use of the full coverage preformed crowns (PCs) to restore the primary dentition has become widely adopted.

The full coronal coverage afforded by PCs makes them indispensable to the paediatric armamentarium, irrespective of the material composition. The preformed metal crown (PMC) was first introduced in 1950 to help restore the primary dentition affected by multi-surface lesions of dental caries, developmental defects, fractures, or extensive wear. PMC are also used after pulp therapy, or as abutments for space maintainers (Engel 1950; Humphrey 1950). The crowns are available in multiple sizes and can be modified chairside by means of crimping, trimming, and contouring, for improving adaptation to the tooth (Seale and Randall 2015). PMCs are considered the gold standard in paediatric restorative materials.

During clinical procedures, contamination of PMCs occurs for several reasons. When the crown of the tooth is prepared, subgingival margin preparation results in minor gingival bleeding. When the dental practitioner estimates the crown size, often several crowns of differing sizes are tried-in before one is chosen. Hence, PMCs will be exposed to the patient’s saliva and blood. Both bodily fluids are potentially infectious. The reprocessing and reuse of dental instruments and devices (e.g., rotary endodontic files or molar orthodontic bands) is acceptable only when industry standards and infection control requirements are met (Chan et al. 2016; Fulford et al. 2003). Adding to this, children are more likely than adults to contract infectious diseases from poor infection control practices due to their immunological naivety (Ygberg and Nilsson 2012). For this reason, if reuse is contemplated, there must be clear guidance around suitable decontamination processes, including the aspects of cleaning, sterilisation, and storage (British Dental Association 2003). The trial-and-error nature of sizing PMCs will predictably result in these being sterilised multiple times. Indeed, the high cost of PMCs is a disincentive for discarding crowns after try-in attempts (Abukabbos et al. 2018). Rates of PMC reuse after sterilisation are estimated to be from 86.4 to 98.92% (El Shehaby 2008; Farhin et al. 2013). This emphasises the need for clear guidance around reprocessing of PMCs that were found to be the wrong size, particularly since these are defined as single use devices.

Rising demands for more aesthetic restorations has led to the emergence of stainless steel preformed crowns that are pre-veneered with a tooth coloured resin material, as well as preformed fabricated from zirconia ceramics (Aiem et al. 2017). Limited data exist on the colour stability or structural integrity of aesthetic PCs. Pre-veneered PMCs have a fracture resistance beyond the average occlusal forces of young children, (Baker et al. 1996; Waggoner and Drummond 2006) although microstructural changes caused by steam sterilisation may alter such properties (Kiran et al. 2015; Yilmaz and Guler 2008). While steam sterilisation remains the most widely used sterilisation technique in dental practice, the high cycle temperatures may disturb the bond between the resin and the stainless steel at the interface of the two materials. In fact, some manufacturers advise chemical disinfection rather than steam sterilisation for aesthetic PCs, to avoid such degradation.

A range of detrimental effects on crown materials can occur during reprocessing. For example, resin materials can be discoloured, absorb water or lose leachable components (Villalta et al. 2006). Steam sterilisation can accelerate the ageing process for zirconia ceramics, with detrimental effects on their colour (Volpato et al. 2016). Processes that lead to crown discolouration are an aesthetic concern and a common reason for complaints (Kupietzky et al. 2003). Practitioners need to be aware of material limitations for PCs and their impact on reprocessing practices. In light of this, a systematic review of the literature on reprocessing of PCs used in paediatric dentistry was undertaken.

## Materials and methods

### Search strategy

The Medline (via EbscoHost), CINAHL (via EbscoHost) and Web of Science databases were searched using the following keyword combinations: (“crowns” or “crown”) AND (“pediatric” or “paediatric” or “child” or “children” or “pedodontic*”) AND (“steril*” or “decontamin*” or “autoclave*” or “disinfect*” or “antisepsis” or “asepsis” or “infection control” or “infection control, dental”). The search was conducted on Dec 6, 2021, without language restrictions. Additional Google hand searches and grey literature searches were conducted using the same keywords to identify literature not accessible via standard database retrievals.

### Screening and selection

Studies reporting reprocessing practices for PCs were identified. After screening of titles and abstracts, the reference lists of applicable studies were examined to identify any additional sources. The studies were imported into Endnote™ software (Clarivate, USA), and duplicate articles removed. The first level of selection was undertaken using titles and abstracts, by applying the inclusion and exclusion criteria outlined in Table 1. Subsequently, full text versions were retrieved, and a second level of selection was performed, using the same criteria. The Joanna Briggs Institute’s critical appraisal checklist for non-randomised experimental studies was used to account for risk of bias (Porritt et al. 2014).

### Review

*Inter-rater reliability agreement:* Inter-rater reliability was calculated between the two independent assessors (T.H. and S.Z) during the data extraction (identification, screening, eligibility, and inclusion) stage (%). Inter-rater reliability agreement for the three stages (selection, comparability, and outcome) was evaluated using Cohen’s kappa in the Statistical Package for the Social Sciences version 24.0 software program (IBM Corp., Armonk, NY, USA).

## Results

### Literature search

**Fig. 1:**
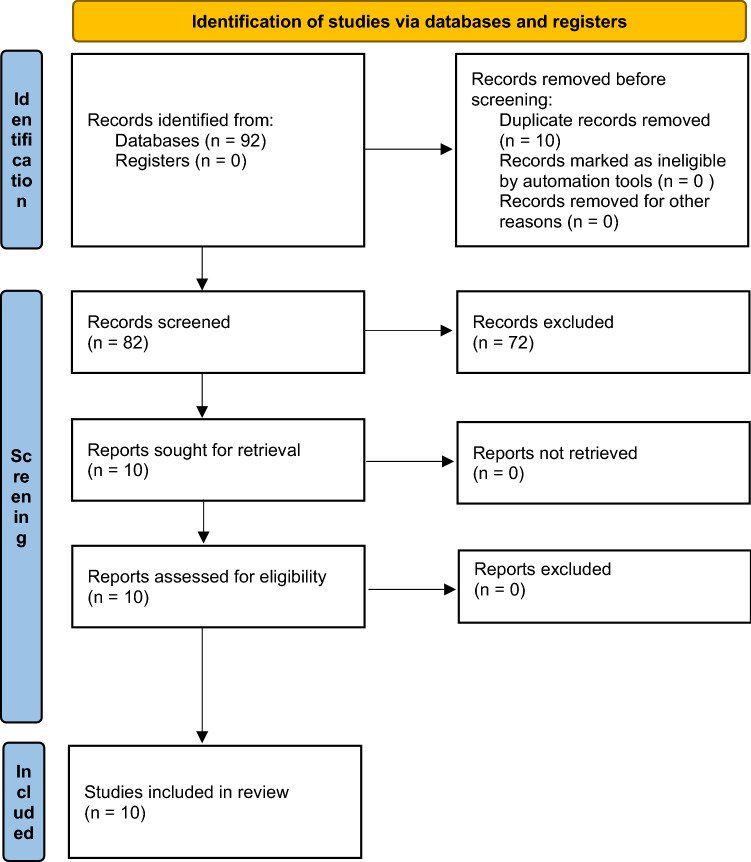
PRISMA (Preferred Reporting Items for Systematic Reviews and Meta-analysis) flowchart of studies

The database search identified 91 papers, with one additional paper recovered via hand searches and from checking reference lists. Of the articles compiled using the search strategy, the first level of selection excluded 72 papers with irrelevant titles or abstracts. Simultaneously, ten duplicate papers were removed. The remaining ten papers met the second level inclusion criteria for the review. The Preferred Reporting Items for Systematic Reviews and Meta-analysis (PRISMA) guidelines flowchart is shown in Fig. 1 (Pate et al. 2021). No papers reported an objectionable risk of bias.

### Inter-rater reliability agreement

The inter-rater reliability agreement between the two assessors was 96.8% (92/94 studies), 96.7% (72/74 studies), 100% (10/10 studies), and 100% (10/10 studies) at the identification, screening, eligibility, and inclusion stages, respectively. Any discrepancies were resolved through discussion until a consensus was reached. Each domain had either a strong to almost perfect inter-agreement reliability between the independent reviewers, with kappa scores of 0.849 (selection), 0.924 (comparability), and 0.941 (outcome).

### Outcomes from the included studies

The included articles for qualitative synthesis focussed on PMCs, pre-veneered PMCs, and zirconia preformed crowns, with nine experimental reports and one survey of clinicians (Table 2). The methods for reprocessing that were investigated included steam sterilisation (autoclaving), chemiclave sterilisation, and chemical disinfection. There were no studies of the differential effects on PCs of the three different levels of chemical disinfection (low, intermediate, and high), nor other methods of sterilisation (such as gamma irradiation, ozone sterilisation or hydrogen peroxide gas plasma sterilisation). The included studies used a range of methods to assess degradation of PCs, with assessments of crowns at the macroscopic level (colour changes, dimensional changes) and surface changes at the microscopic level (such as crazing and corrosion) being the most common (Table 2). Only one study explored changes in elemental composition. Studies were broadly divided into two groups according to whether the evaluated outcomes were physical or biological in nature.

Studies in Group 1 assessed changes to material properties such as colour stability or microstructural characteristics (e.g., crazing, fracturing, contour alterations). Pre-veneered PMCs showed colour changes in the composite resin component, while the colour of zirconia crowns was not affected by steam sterilisation or by chemical disinfection. Aesthetic alterations to pre-veneered PMCs required combined chemical disinfection with steam autoclaving (Hogerheyde et al. 2021). Similarly, chemiclave sterilisation with formaldehyde at 132 °C for 20 min inducing negative colour changes to pre-veneered PMCs (Wickersham et al. 1998). The pre-veneered PMCs propriety resin coatings as well as variable sterilisation parameters yielded inconsistent material changes (Yilmaz and Guler 2008). Stainless steel PMCs and pre-veneered PMCs were reported to develop crazing after multiple cycles of steam sterilisation, but the measures used to report this varies between studies, as well as the number of cycles. Microstructural changes were clinically undetectable requiring microscopic enhancement (Yilmaz and Guler 2008). Scanning electron microscopy (SEM) identified crazing and contour alterations of the vestibular surface of aesthetic PCs. Similar results were reported using stereomicroscope magnification with PMCs exhibiting the highest degree of structural variations (Kiran et al. 2015).

There were only two studies in Group 2 where the efficacy of reprocessing protocols were assessed. Shelburne et al. (2003) reported a greater than 6 log reduction in adherent *Streptococcus mutans* bacteria with steam sterilisation of pre-veneered PMCs. Darshan et al. (2019) demonstrated that contamination on stainless steel PMC that had been tried into the mouth was reduced effectively by steam sterilisation. The effectiveness of decontamination methods on PMCs was assessed via colony counts of *Streptococcus mutans*, *Staphylococcus aureus*, and *Escherichia coli* on selective media plates. In both studies, steam sterilisation was superior to treatment with sodium hypochlorite at 5% or 10%. Neither study was concerned with the efficacy of decontamination protocols on viral or fungal contaminants.

## Discussion

Dentists have a responsibility to maintain high standards of infection control for patient care. The constant progression of industry guidelines to address infection control concerns has led to better outcomes for patients and staff alike. For example, the decontamination protocols used for orthodontic appliances and dental prostheses have received considerable scrutiny (Benson and Douglas 2007; Benyahia et al. 2012; Chassot et al. 2006). Whether practitioners can safety recirculate sterilised PCs is significantly underreported.

The current review revealed that PCs are susceptible to commonly utilised infection control practices. Indeed, several studies identified vestibular crazing and colour instability in PCs following steam sterilisation and/or chemical disinfection (Hogerheyde et al. 2021; Kiran et al. 2015; Padmanabh and Patel 2021; Wickersham et al. 1998; Yilmaz and Guler 2008). However, the most stable PC material was zirconia ceramics (Pate et al. 2021).

PCs are routinely placed by dentists to protect a compromised dentition (Kindelan et al. 2008). Most manufacturers define PCs as single use devices, however, discarding contaminated crowns is financially impractical for dental surgeries. Practitioners need clarification on whether contamination with bodily fluids constitutes treatment. Even the sterility of unopened PCs cannot be guaranteed. Studies have reported nonsterile endodontic files and dental burs supplied by the manufacturer (Kumar et al. 2015). Thus, the risk of environmental contaminants such as bacterial spores is highly plausible.

Inconsistent guidelines on PC reuse are problematic for practitioners with potential medicolegal issues. Researchers surveyed a cohort of paediatric dentists to determine the extent of material recirculation and infection control protocols. The overwhelming majority of respondents (98.92%) reuse PCs, with 57.65% using steam sterilisation as part of the decontamination procedure (Farhin et al. 2013). PCs are classified as a critical item, which requires sterilisation parameters to be met. The Centers for Disease Control and Prevention (CDC) recommends steam autoclaving for critical items as the gold standard (Kohn et al. 2003). Meanwhile heat sensitive items can be sterilised using ethylene oxide or hydrogen peroxide gas plasma (Kanemitsu et al. 2005; Mendes et al. 2007). The survey results reveal a significant portion of practitioners reusing a critical item that has not met international standards for medical treatment. As such, the basic requirements of infection control and patient care are not being met. The inconsistent methods of decontamination as well as wide-ranging infection control learning resources among respondents led the authors to recommend the need for clarity on PC sterilisation guidelines.

The benefits of reducing bacterial populations by steam autoclaving cannot be overstated. An in vivo study comparing decontamination methods on tried-in PMCs found autoclaving at 121 °C, 15 psi for 15 min significantly reduced the colony counts, compared with 5% sodium hypochlorite (5 min), 5% glutaraldehyde (5 min), 70% isopropyl (1 min), or glass bead groups (Darshan et al. 2019). The insufficient reduction in bacterial counts for rapid chairside sterilisation (i.e., sodium hypochlorite, glutaraldehyde, etc.) led authors to conclude that autoclaving was paramount for effective sterility. Similarly, the efficacy of infection control practices on contaminated pre-veneered PMCs using 70% ethanol, 10% sodium hypochlorite, or steam autoclaving has been studied (Shelburne et al. 2003). The adherent *Streptococcus mutans* bacteria was then cultured using agar growth plates to assess the degree of bacterial reduction. All protocols reduced levels of bacteria by at least six logs. However, a significant number of variables can affect the outcome. Incomplete sterilisation can occur when any disparities in temperature, contact time, or concentration of liquid agents exists. For protocols that omit steam autoclaving the degree and consistency of microbial elimination is quite variable (Wickersham et al. 1998). Whilst some manufacturers recommend cold sterilisation for heat sensitive PC materials, the techniques need to be carefully calibrated (Benyahia et al. 2012).

Various PC materials may respond differently to infection control practices. The need for customised decontamination protocols may be essential for some crown materials. For example, whether PCs are vulnerable to steam autoclaving is debatable. Studies have reported conflicting evidence with PMCs showing evidence of crazing and microstructural changes (Kiran et al. 2015; Yilmaz and Guler 2008). A comparative study evaluating decontamination methods on PCs observed crazing and contour alterations following steam autoclaving (Yilmaz and Guler 2008). However, changes were not observable using stereomicroscope imaging. SEM analysis was necessary to discern notable microstructural changes. As such, practitioners may unknowingly recirculate PCs with compromised structural properties. To date the effects on clinical performance are unknown. The authors of another study recommended using an aldehyde-free high grade disinfectant to decontaminate PCs that have exposed to bodily fluids (Yilmaz and Guler 2008).

Additional studies noted deleterious effects for autoclaving at 121 °C, 15 psi, 20 min and 132 °C, 30 psi, 8 min with crazing in one-third to one-half of PMCs (Kiran et al. 2015). Such topographical irregularities may yield a more retentive surface for dental plaque as well as promoting unwanted corrosion (Marentes 2018; Wickersham et al. 1998). Others identified no microstructural changes to PMCs after five autoclave cycles (Marentes 2018). The authors postulated that material variations between manufacturers may account for corrosion sites in the trough regions. In addition, a recent study investigating the effects of infection control practices (i.e., fast/slow steam autoclaving, ultrasonic bath) on the physical–mechanical properties of preformed crowns found no evidence of fractures or colour changes (Padmanabh and Patel 2021). However, maximum crazing was seen in PMCs. The materials tested included PMCs, pre-veneered PMCs, and zirconia crowns.

A greater awareness of childhood psychology as well as demand from parents has led practitioners to offer aesthetic PCs. It has been shown that disinfectants can modify the colour stability of denture base resins (Bensel et al. 2018). Similarly, persistent thermocycling of monolithic zirconia can produce significant colour and translucency changes (Koseoglu et al. 2019). However, limited data have been published on the impact of infection control practices on aesthetics of crowns. For example, neither autoclaving nor glutaraldehyde sterilisation had minimal effect on colour parameters for pre-veneered PMCs (Wickersham et al. 1998). However, the same PCs underwent negative colour changes when subject to chemiclave sterilisation. The present authors reported that pre-veneered PMCs showed colour variations with loss of luminosity after reprocessing (Hogerheyde et al. 2021). Thus, clinical awareness of material compatibility is paramount to limit unwanted aesthetic variations. A recent study evaluated the aesthetic characteristics of four brands of zirconia crowns after autoclave and cold sterilisation (Pate et al. 2021). The authors concluded no significant differences in colour stability, gloss, or translucency between groups. Similar results were described by the current authors with zirconia crowns unaffected by reprocessing protocols.

The systematic review was limited by experimental disparities such as sterilisation parameters or manufactures used. Although considerable research has been conducted into the decontamination practices on restorative dental materials, the effect on paediatric materials is substantially unreported. The review herein was constrained by a lack of robust in vivo studies, indicating the need for widening the analysis to include non-paediatric dental materials. However, the assembled literature offers a blueprint for future studies by identifying consistent material deficiencies such as colour instability and microstructural variations. Future studies need to focus on identifying the specific conditions (i.e., temperature, cycle number, etc.) responsible for such material degradation. The maintenance and operational parameters of sterilisation equipment is paramount for effective sterility. For instance, challenge tests such as biological indicators require specific conditions such as temperature, pressure, and time to be met (Palenik et al. 1999). If practitioners chose to recirculate decontaminated materials, then utmost confidence in the sterilisation process is vital.

## Conclusions

Considering any limitations of the present review it has been shown that:

- Manufacturers need to better define the sterilisation parameters and reuse for the supplied material.
- A consensus needs to be reached whether practitioners can legitimately recirculate decontaminated PCs. Manufactures have started to address concerns of cross contamination by offering try-in moulds that can be autoclaved at high temperatures.
- Recent advances in hydrogen peroxide gas plasma sterilisation offers dental surgeries alternative technologies to process contaminated PCs. The reduced cycle time and temperature means that heat sensitive materials can be effectively sterilised.

**Table 1:** Inclusion and exclusion criteria adopted in the literature search

Inclusion criteria	Exclusion criteria
**P:** Participants: any gender, up to 18 years old	Participants: adults (older than 18 years)
**I:** Interventions: decontamination of preformed paediatric crown materials	Custom-built adult crowns
**C:** Comparison: material recirculation, decontamination protocols	Reviews, letters to the editor, editorials
**O:** Outcomes: adverse material outcomes from decontamination practices, efficacy against microorganisms	
**Study design:** randomised and non-randomised control trials, cohort studies, case reports, surveys, case–control studies, in vitro and in vivo studies	
Preformed paediatric crowns—zirconia, PMC, pre-veneered PMC, chrome steel, SSCs	
Publication year: no restrictions Language: no restrictions

**Table 2:** Characteristics of the included studies

Author (year)	Design	Crown materials	Decontamination methods	Measure	Key findings
Wickersham et al. (1998)	NRT	Pre-veneered PMC (*n* = 35)	Steam autoclaving Chemiclave sterilisation Chemical disinfection	Colour analysis Surface changes Fracture resistance	Pre-veneered PMCs recorded negative colour changes following chemiclave sterilisation. Chemical disinfection with 2% glutaraldehyde
Shelburne et al. (2003)	NRT	Pre-veneered PMC (*n* = 5)	Steam autoclaving Chemical disinfection	Microbial colony counts	All protocols reduced adherent *Streptococcus mutans* bacteria by at least 6 logs. The authors concluded that steam autoclaving, 10% hypochlorite, and 70% ethanol were suitable for clinical use
Yilmaz and Guler (2008)	NRT	Polycarbonate Pre-veneered PMC (*n* = 80)	Steam autoclaving Chemical disinfection	Crazing Contour alterations Fracturing Surface changes	Steam autoclaving caused significant crazing and contour alterations on all crown materials under SEM. The stereomicroscope imaging was unable to detect vestibular changes. The authors recommended an aldehyde-free disinfectant as the preferred method for decontaminating crowns
Farhin et al. (2013)	DS	- (*n* = 95; survey participants)	-	Rates of recirculation Reasons for reuse Decontamination protocols Infection control learning resources	Most dentists reused decontaminated PMCs following try-in attempts. No consistent decontamination method was used between respondents
Kiran et al. (2015)	NTR	PMC (*n* = 15)	Steam autoclaving	Crazing	PMCs exhibited crazing after steam autoclaving at various exposure times and temperatures
Marentes (2018)	NRT	PMC (*n* = 18)	Steam autoclaving Chemical disinfection	Elemental composition Crazing Fracturing Surface changes Pitting corrosion	Multiple autoclave cycles had no adverse effect on PMC integrity or microstructural properties. Manufacturing defects on PMC surfaces potentially increases unwanted corrosion
Darshan et al. (2019)	NRT	PMC (*n* = 140)	Steam autoclaving Chemical disinfection	Microbial colony counts	Steam autoclaving was most effective at reducing microbes on tried-in PMCs; followed by 5% glutaraldehyde and 5% sodium hypochlorite. Chairside disinfectants were not recommended as reliable decontamination methods for patient safety
Pate et al. (2021)	NRT	Zirconia ceramic (*n* = 64)	Steam autoclaving Chemical disinfection	Colour stability	No clinically significant changes in colour stability were noted for zirconia crowns, irrespective of the method used
Padmanabh and Patel (2021)	NRT	PMC Pre-veneered PMC Zirconia ceramic (*n* = 60)	Steam autoclaving Chemical disinfection	Colour change Crazing Dimensional stability Fracturing	No fracturing or colour change was recorded for slow/fast autoclaving or chemical disinfection. PMCs exhibited maximum crazing, while zirconia crowns were unaffected
Hogerheyde et al. (2021)	NRT	PMC Pre-veneered PMC Zirconia ceramic (*n* = 30)	Chemical disinfection Steam autoclaving	Colour stability Surface changes Elemental composition	Pre-veneered crowns showed colour variations and loss of luminosity after reprocessing. PMCs and zirconia crowns were unaffected by chemical disinfection or steam autoclaving

*NRT* quantitative non-randomised trial, *DS* quantitative descriptive study

## Data Availability

The data used for this research article can be made available upon request to the authors.
